# Socioeconomic status, financial hardship and measured obesity in older adults: a cross-sectional study of the EPIC-Norfolk cohort

**DOI:** 10.1186/1471-2458-13-1039

**Published:** 2013-11-04

**Authors:** Annalijn I Conklin, Nita G Forouhi, Marc Suhrcke, Paul Surtees, Nicholas J Wareham, Pablo Monsivais

**Affiliations:** 1 MRC Epidemiology Unit, Institute of Metabolic Science, Addenbrooke’s Hospital, University of Cambridge, Hills Road, Box 285, Cambridge CB2 0QQ, UK; 2 UK Clinical Research Collaboration Centre for Diet and Activity Research (CEDAR), Institute of Public Health, Forvie Site, Robinson Way, Box 296, Cambridge CB2 0SR, UK; 3 Faculty of Medicine and Health Sciences, University of East Anglia, Norwich NR4 7TJ, UK; 4 Department of Public Health and Primary Care, Institute of Public Health, University of Cambridge, Cambridge CB1 8RN, UK

**Keywords:** Body mass index, Waist circumference, Anthropometry, Socioeconomic status, Financial hardship, Elderly, Healthy ageing, EPIC-Norfolk

## Abstract

**Background:**

Socioeconomic status is strongly associated with obesity. Current economic circumstances are also independently associated with self-reported weight status in Finnish civil servants. We aimed to examine three types of financial hardship in relation to measured general and central obesity in a general population of older adults, while considering conventional socioeconomic indicators.

**Methods:**

Data from 10,137 participants (≥50 years) in the EPIC-Norfolk cohort who responded to a postal Health and Life Experiences Questionnaire (1996–2000) and attended a clinical assessment (1998–2002). Multivariable logistic regression models assessed likelihood of general obesity (BMI ≥30 kg/m^2^) and central obesity (women: ≥88 cm; men: ≥102 cm) calculated from measured anthropometrics.

**Results:**

Obesity prevalence was consistently patterned by standard socioeconomic indicators, with over-50s in the lowest social class being twice as likely to be obese than those in the highest class (women OR 2.10 [CI95: 1.41—3.13]; men OR 2.36 [1.44—3.87]). After adjustment for socioeconomic status, reporting *having less than enough money for one’s needs* (compared to more than enough) was associated with obesity in women (OR 2.04 [1.54—2.69]) and men (OR 1.83 [1.34—2.49]). Similar associations were demonstrated between obesity and *always or often not having enough money for food/clothing* (women OR 1.40 [1.03—1.90]; men OR 1.81 [1.28—2.56]), compared to reporting this never occurred. The strongest independent associations were seen for obesity and reported greatest level of *difficulty paying bills* (women OR 2.20 [1.37—3.55]; men 2.40 [1.38—4.17]), compared to having no difficulties. Findings for central obesity were slightly higher in women and lower in men.

**Conclusions:**

Obesity in British over-50s was more likely in study participants who reported greater financial hardship, even after education, social class and home ownership were taken into account. Public health policies need to consider the hitherto neglected role of financial hardship in older people, especially difficulty paying bills, as part of strategies to prevent or reduce obesity.

## Background

Obesity is one of the biggest public health challenges faced by high income countries, accounting for a large and growing disease burden (cardiovascular disease, type 2 diabetes, some cancers) and imposing substantial cost burden on both the healthcare system and society at large [1-3]. Both general (weight status) and central (excess abdominal fat) obesity significantly increase a person’s risk of, for example, cardiometabolic conditions [4]. Obesity is strongly and inversely related to socioeconomic status (SES) as measured by conventional indicators of education or social class, particularly in the case of women [5-7]. But, conventional SES has been acknowledged as insufficient to understand how a person’s economic situation is associated with their body mass index (BMI) or waist circumference (WC) [8], and differences in obesity by education or income are not consistently observed in UK adults [9].

A limitation with conventional SES indicators is that they do not fully capture people’s material circumstances and spending power, whereas everyday financial troubles may be a stronger antecedent to obesity than income, occupational status, or education [8,10]. Wider research on poverty has highlighted the added value of material hardship measures to concepts of inequality [11]. Evidence from two occupational cohorts supports the notion that financial hardship reflects a distinct set of economic factors that independently impacts health beyond the reported influence exerted by conventional SES [8,10,12-15]. Another study found an association between financial hardship and smoking even within high-income groups [16]. To date, one longitudinal study of US adolescents explored separate types of financial hardship and found suggestive evidence that having trouble paying bills may be related to obesity in women, but not men [17].

Financial hardship is closely correlated, but not interchangeable, with conventional SES and therefore deserves specific attention [18]. Since individuals at all income levels can experience financial hardship with consequent health effects, the implications for public health and policy are that income (or other standard SES indicators) should not be seen as the sole criteria for targeting interventions [12,18-20]. One of the largest drains on disposable income, especially in older people, is paying bills and affording adequate food and clothing [21], and yet, health research and policy has tended to neglect current financial difficulties as a unique economic domain determining health. Consequently, evidence remains limited on the role of financial hardship in obesity and whether separate types of hardship differ in their associations with obesity, particularly among older adults who comprise a growing population [8,19,22]. Older adults with greater hardships may purchase less food and have lower weights due to fewer calories; however, they may also purchase cheaper food high in energy density which could contribute to excess weight.

We therefore investigated the sex-specific associations between three types of self-reported financial hardships and obesity measured objectively in adults aged 50 and older. We hypothesised that greater levels of financial hardship may be associated with greater odds of obesity, overall and centrally, with sex differences in magnitude of associations. We further hypothesised that associations would remain significant after adjusting for conventional socioeconomic status.

## Methods

### Study population

We used data collected as part of the EPIC-Norfolk prospective cohort study—a component of the European Prospective Investigation of Cancer (EPIC) study in 10 European countries [23]. As we were interested in adults near the end of working life and beyond to place findings in a healthy ageing context, we included over-50s (n = 20,274) from the population-based cohort who were recruited from age-sex registers of general practices and who attended a first health check at entry (1993–97). At entry, over-50s were similar to the total cohort (n = 25,639) in terms of health behaviours and other socio-demographic factors. Ethnicity of 99.7% of EPIC-Norfolk participants was of white origin.

Financial hardship was assessed using the postal “Health and Life Experiences Questionnaire” (HLEQ) (1996–2000) designed to assess social and psychological circumstances [24,25]. Completed responses from over-50s ranged between 17,953 and 17,998. Outcome data on BMI (n = 11,982) and WC (n = 12,000) were measured objectively during a second clinical assessment (1998–2002). Our available sample therefore included over-50s who responded to financial hardship questions, had covariates and follow-up anthropometry (range: 10,113—10,137) with 99% complete data (see Additional file 1: Figure S1). The sample was similar in characteristics and lifestyle to responders in the full cohort, and over-50s not responding to hardship questions were similar to cohort non-responders. All volunteers gave written informed consent and the study was approved by the Norwich district ethics committee.

### Measures

#### Financial hardship and conventional socioeconomic indicators

Financial hardship was measured by three self-reported questions in line with Pearlin’s list of chronic strains as used in similar studies [12,20]. Questions covered sufficiency of money to meet needs (more than enough, just enough, less than enough), frequency of not having enough money to afford adequate food or clothing (5 responses, range 'always’ to 'never’), and difficulty paying bills (6 responses, range 'very great’ to 'none’). Responses 'always’ and 'often’ , or 'great’ and 'very great’ , were combined for analysis due to low numbers in the bottom two categories.

We analysed three conventional indicators of SES. Education level (no qualification, O-level (16 years), A-level (18 years), degree (>18 years)) and occupation were self-reported at cohort entry, with occupation used to classify participants into six hierarchical categories of the Registrar General’s classification scheme of social class (professional, managerial and technical, skilled non-manual, skilled manual, partly skilled, and unskilled). Social class in women was based on her partner’s occupation (68%) unless it was unclassified, missing, or they were single and then her own occupation was used [26]. We also employed a measure of housing tenure (home-owner, public renting, private renting) as a conventional SES proxy, given that previous research has documented the utility of home ownership as a measure of wealth in older populations [27].

#### Obesity

Trained nurses used standardised protocols to measure weight, height, and WC of all participants attending the second clinic assessment, as reported elsewhere [26,28]. BMI was calculated as weight in kilograms divided by the square of height in metres. Participants who had a BMI ≥ 30 kg/m^2^ were classified as obese overall. Central obesity was calculated using sex-specific threshold criteria for WC: women with WC ≥ 88 cm and men with WC ≥ 102 cm were classified as centrally obese.

#### Socio-demographic variables

Participants self-reported smoking status (current, former, never), marital status (married/living as married, single, widowed, separate, divorced), and general health status (excellent, good, moderate, poor), at second health check (1998–2002). Regular car use (yes/no) was self-reported in the Environment and Physical Activity questionnaire (EPAQ2) (1998–2000). Socio-demographic variables for date of birth (continuous age) and sex were measured at cohort entry.

### Data analysis

Descriptive statistics summarised socio-demographic characteristics (sex, education, social class, home-ownership, car use, health status, smoking status, marital status), and crude prevalence of obesity across financial hardship levels. We used a correlation matrix to examine inter-relationships among the financial hardship indicators after recoding two indicators into three levels: frequency of not having enough money for food or clothing (never; sometimes/seldom; often/always), and difficulty paying bills (none; very little/slight; some/great/very great). Odds ratios of prevalent obesity for the six socioeconomic indicators were examined by fitting logistic regression models *a priori* sex-stratified and adjusted for age, smoking status and marital status. As known confounders, each is associated with socioeconomic factors and independently with obesity [29-32].

For each categorical measure of financial hardship, we fitted sequential logistic regression models to base models (sex-stratified and adjusted for age, smoking and marital status); first by education, followed by occupational social class, and then housing tenure. The final model for each hardship indicator mutually adjusted for all conventional SES and covariates and therefore the remaining sex-specific odds ratios for general and central obesity were interpreted as independent associations of the financial hardship variable in question.

In secondary analyses, we further adjusted for concurrent lifestyle variables: total energy intake (Kcal), total alcohol consumption (units/week), and physical activity and energy expenditure (PAEE) score. Self-reported total energy and alcohol intake were assessed by food frequency questionnaire [33]; and PAEE by the EPAQ2 questionnaire, previously validated against individually calibrated heart rate against energy expenditure [34]. For women, we also adjusted for menopause and HRT status in secondary models.

Statistical analyses were performed separately for women and men using Stata 12.1 [35]. Results are presented as odds ratios (ORs) and 95 percent confidence intervals (95% CIs).

## Results

The mean age of participants was 62.5 years (SD 7.5) with 54% of the sample made up of women. A majority (81%) reported being in good or excellent general health, and 51% were ever smokers. For the whole sample, 11% were educated to degree-level; 14% of men and 9% of women were educated to this level. Professional (class I), and managerial and technical (class II), occupations comprised 42% of the sample; few women (4%) and men (3%) had unskilled occupations. Mean BMI was 27 kg/m^2^ (SD 3.3) in men, and 26.8 kg/m^2^ (SD 4.4) in women; 16% of men and 20% of women were classified as obese overall. Women’s average WC was 82.9 cm (SD 10.6) and men’s was 96.7 cm (SD 9.6); 29% of women and 27% of men were classified as being centrally obese.

There was a close inter-relationship between the three measures of self-reported financial hardship and other socio-demographic measures (see Additional file 1: Table S2). We found that the three financial hardship measures were moderately related to each other. The indicator having enough money for needs shared 23% and 26% of its variability with the indicators frequency of not having enough money for food or clothing (r = 0.48) and difficulty paying bills (r = 0.51), respectively. Frequency of not having enough money for food or clothing shared 38% of its variability with difficulty paying bills (r = 0.62).

### Conventional SES indicators and odds of obesity

There was a clear pattern of association between lower levels of social class, education, and housing tenure and general and central obesity in sex-specific models adjusted for covariates (Table 1). The lowest social class (V) was significantly associated with greater odds of general obesity in women (OR 2.10; 1.41—3.13) and men (OR 2.36; 1.44—3.87) aged 50 and over. Similar sex-specific associations were observed between social class and central obesity, but reached significance only in women. Women and men who reported having no educational qualification were more likely to be obese centrally and overall, although odds ratios were larger in men for both outcomes. Similarly, obesity was more likely in women and men who reported renting public or private accommodation (compared with owning); magnitudes were largest for general obesity in men who rented accommodation.

**Table 1:** Odds ratios of general and central obesity in women and men (≥50 years) across levels of social class, education, and housing tenure in the EPIC-Norfolk cohort

	**General obesity**		**Central obesity**	
	**Women**	**Men**	**Women**	**Men**
***Social Class***	*(n* = *6320)*	*(n* = *5277)*	*(n* = *6327)*	*(n* = *5286)*
I	1.00	1.00	1.00	1.00
II	1.29 (0.96, 1.72)	1.33 (0.96, 1.84)	1.11 (0.88, 1.41)	1.08 (0.85, 1.37)
III–non manual	1.23 (0.90, 1.66)	1.57 (1.09, 2.26)	0.99 (0.77, 1.28)	1.13 (0.86, 1.49)
III–manual	1.49 (1.10, 2.02)	1.53 (1.09, 2.14)	1.10 (0.85, 1.41)	1.03 (0.80, 1.34)
IV	2.10 (1.53, 2.86)	1.36 (0.94, 1.96)	1.41 (1.08, 1.84)	1.10 (0.83, 1.45)
V	2.10 (1.41, 3.13)	2.36 (1.44, 3.87)	1.57 (1.11, 2.22)	1.47 (0.97, 2.24)
***Education***	*(n* = *6464)*	*(n* = *5353)*	*(n* = *9531)*	*(n* = *6327)*
Degree	1.00	1.00	1.00	1.00
A-level	1.16 (0.92, 1.46)	1.48 (1.28, 2.15)	1.12 (0.92, 1.36)	1.17 (0.97, 1.42)
O-level	1.33 (1.01, 1.74)	1.27 (0.90, 1.78)	1.07 (0.84, 1.36)	1.27 (0.98, 1.65)
No qualification	1.59 (1.27, 1.99)	1.66 (1.28, 2.15)	1.38 (1.14, 1.67)	1.42 (1.16, 1.73)
***Housing tenure***	*(n* = *5727)*	*(n* = *4706)*	*(n* = *5734)*	*(n* = *4712)*
Owner	1.00	1.00	1.00	1.00
Renting, private	1.49 (1.02, 2.17)	1.85 (1.20, 2.85)	1.41 (1.00, 1.99)	1.43 (0.97, 2.11)
Renting, public	1.49 (1.15, 1.92)	1.70 (1.22, 2.35)	1.66 (1.32, 2.08)	1.69 (1.27, 2.24)

Sex-specific odds ratios (95% confidence intervals) obtained by multivariable logistic regression analysis adjusting for age, marital status and smoking status. General obesity (BMI ≥ 30 kg/m^2^); Central obesity (women: waist circumference, WC ≥ 88 cm; men: WC ≥ 102 cm).

Further adjustment for total energy intake, physical activity and alcohol intake attenuated or made little difference to most of the associations between conventional SES indicators and obesity (see Additional file 1: Table S3). Addition of menopause and HRT status made no difference to overall magnitude or direction of findings for women (data not shown).

### Financial hardship and odds of obesity

In an analysis adjusting for age, smoking status, and marital status, all three measures of financial hardship were strongly associated with obesity in both sexes (Model A, Table 2). The magnitude of association was greater than that seen for the more traditional measures of SES. In general the measures of association were similar in men and women.

**Table 2:** Odds ratios of general obesity across levels of financial hardship in women and men (≥50 years) in the EPIC-Norfolk cohort

	**Women**			
	***Model A***	***Model B: A + education***	***Model C: B + social class***	***Model D: C + housing tenure***
***Enough money for needs***				
More than enough	1.00	1.00	1.00	1.00
Just enough	1.50 (1.24, 1.81)	1.39 (1.15, 1.69)	1.36 (1.12, 1.65)	1.34 (1.10, 1.64)
Less than enough	2.56 (1.98, 3.33)	2.32 (1.78, 3.03)	2.20 (1.68, 2.88)	2.04 (1.54, 2.69)
***Frequency of not enough money***				
Never	1.00	1.00	1.00	1.00
Seldom	1.41 (1.21, 1.66)	1.38 (1.18, 1.62)	1.36 (1.16, 1.60)	1.35 (1.14, 1.59)
Sometimes	1.64 (1.36, 1.98)	1.57 (1.30, 1.90)	1.53 (1.26, 1.85)	1.44 (1.18, 1.76)
Often/Always	1.68 (1.26, 2.25)	1.57 (1.18, 2.10)	1.54 (1.15, 2.06)	1.40 (1.03, 1.90)
***Difficulty paying bills***				
None	1.00	1.00	1.00	1.00
Very little	1.38 (1.18, 1.62)	1.37 (1.16, 1.60)	1.33 (1.13, 1.56)	1.32 (1.12, 1.56)
Slight	1.78 (1.41, 2.25)	1.75 (1.39, 2.21)	1.76 (1.39, 2.23)	1.67 (1.32, 2.13)
Some	2.08 (1.66, 2.61)	1.99 (1.59, 2.50)	1.92 (1.53, 2.42)	1.81 (1.43, 2.30)
Great/Very great	2.52 (1.60, 3.98)	2.37 (1.50, 3.75)	2.38 (1.50, 3.78)	2.20 (1.37, 3.55)
	**Men**			
	***Model A***	***Model B: A + education***	***Model C: B + social class***	***Model D: C + housing tenure***
***Enough money for needs***				
More than enough	1.00	1.00	1.00	1.00
Just enough	1.28 (1.02, 1.59)	1.22 (0.97, 1.53)	1.18 (0.94, 1.48)	1.17 (0.93, 1.47)
Less than enough	2.13 (1.59, 2.85)	2.00 (1.49, 2.69)	1.93 (1.43, 2.61)	1.83 (1.34, 2.49)
***Frequency of not enough money***				
Never	1.00	1.00	1.00	1.00
Seldom	1.32 (1.09, 1.60)	1.30 (1.07, 1.57)	1.29 (1.06, 1.56)	1.30 (1.07, 1.59)
Sometimes	1.30 (1.01, 1.66)	1.26 (0.98, 1.62)	1.24 (0.97, 1.60)	1.15 (0.88, 1.49)
Often/Always	2.04 (1.47, 2.84)	1.98 (1.42, 2.76)	1.93 (1.38, 2.71)	1.81 (1.28, 2.56)
***Difficulty paying bills***				
None	1.00	1.00	1.00	1.00
Very little	1.05 (0.87, 1.28)	1.04 (0.86, 1.27)	1.04 (0.85, 1.26)	1.02 (0.84, 1.24)
Slight	1.18 (0.87, 1.60)	1.17 (0.86, 1.59)	1.17 (0.86, 1.58)	1.13 (0.83, 1.55)
Some	1.61 (1.21, 2.15)	1.58 (1.18, 2.11)	1.54 (1.15, 2.06)	1.39 (1.02, 1.88)
Great/Very great	2.48 (1.45, 4.26)	2.43 (1.42, 4.18)	2.51 (1.46, 4.34)	2.40 (1.38, 4.17)

Sex-specific odds ratios (95% confidence intervals) for general obesity (BMI ≥ 30 kg/m^2^) obtained by multivariable logistic regression analysis adjusting for age, marital status and smoking status. Final numbers (Model D) of women and men, respectively, for money (n = 5 526; n = 4 588); frequency of not enough money (n = 5 536; n = 4 591); difficulty paying bills (n = 5 542; n = 4 596).

A somewhat different pattern was observed for associations with central obesity which tended to be stronger in women than men (Model A, Table 3). Adjusting for education, social class, and housing tenure attenuated the associations between financial hardship and odds of general and central obesity (Model D, Table 2 and Table 3).

**Table 3:** Odds ratios of central obesity across levels of financial hardship in women and men (≥50 years) in the EPIC-Norfolk cohort

	**Women**			
	***Model A***	***Model B: A + education***	***Model C: B + social class***	***Model D: C + housing tenure***
***Enough money for needs***				
More than enough	1.00	1.00	1.00	1.00
Just enough	1.55 (1.31, 1.83)	1.48 (1.25, 1.75)	1.50 (1.26, 1.78)	1.50 (1.26, 1.78)
Less than enough	2.51 (1.99, 3.18)	2.37 (1.86, 3.00)	2.32 (1.82, 2.97)	2.16 (1.68, 2.78)
***Frequency of not enough money***				
Never	1.00	1.00	1.00	1.00
Seldom	1.33 (1.15, 1.53)	1.30 (1.13, 1.50)	1.29 (1.12, 1.49)	1.29 (1.11, 1.49)
Sometimes	1.49 (1.26, 1.77)	1.45 (1.22, 1.72)	1.44 (1.21, 1.71)	1.38 (1.15, 1.65)
Often/Always	1.76 (1.36, 2.29)	1.69 (1.30, 2.19)	1.65 (1.26, 2.15)	1.51 (1.15, 1.99)
***Difficulty paying bills***				
None	1.00	1.00	1.00	1.00
Very little	1.39 (1.21, 1.60)	1.38 (1.20, 1.59)	1.36 (1.18, 1.56)	1.36 (1.17, 1.57)
Slight	1.57 (1.26, 1.94)	1.55 (1.25, 1.92)	1.57 (1.26, 1.95)	1.50 (1.20, 1.87)
Some	1.74 (1.41, 2.15)	1.69 (1.37, 2.09)	1.68 (1.36, 2.08)	1.59 (1.28, 1.99)
Great/Very great	2.66 (1.73, 4.08)	2.55 (1.66, 3.92)	2.46 (1.59, 3.82)	2.34 (1.49, 3.67)
	**Men**			
	***Model A***	***Model B: A + education***	***Model C: B + social class***	***Model D: C + housing tenure***
***Enough money for needs***				
More than enough	1.00	1.00	1.00	1.00
Just enough	1.19 (1.00, 1.42)	1.14 (0.96, 1.36)	1.16 (0.97, 1.39)	1.14 (0.95, 1.37)
Less than enough	1.76 (1.38, 2.25)	1.66 (1.30, 2.13)	1.69 (1.31, 2.17)	1.64 (1.26, 2.12)
***Frequency of not enough money***				
Never	1.00	1.00	1.00	1.00
Seldom	1.32 (1.13, 1.55)	1.30 (1.11, 1.52)	1.30 (1.11, 1.53)	1.30 (1.11, 1.53)
Sometimes	1.33 (1.08, 1.63)	1.29 (1.05, 1.58)	1.30 (1.06, 1.60)	1.26 (1.01, 1.56)
Often/Always	1.62 (1.20, 2.19)	1.58 (1.17, 2.13)	1.57 (1.16, 2.13)	1.51 (1.11, 2.07)
***Difficulty paying bills***				
None	1.00	1.00	1.00	1.00
Very little	1.14 (0.98, 1.33)	1.13 (0.97, 1.32)	1.14 (0.98, 1.34)	1.13 (0.96, 1.32)
Slight	1.25 (0.97, 1.61)	1.24 (0.97, 1.60)	1.25 (0.97, 1.61)	1.24 (0.96, 1.61)
Some	1.53 (1.19, 1.96)	1.50 (1.17, 1.93)	1.47 (1.14, 1.90)	1.35 (1.04, 1.77)
Great/Very great	1.48 (0.88, 2.49)	1.45 (0.86, 2.44)	1.53 (0.90, 2.59)	1.49 (0.87, 2.54)

Sex-specific odds ratios (95% confidence intervals) obtained by multivariable logistic regression analysis adjusting for age, marital status and smoking status. Central obesity for women (waist circumference, WC ≥ 88 cm) and for men (WC ≥ 102 cm). Final numbers (Model D) of women and men, respectively, for money (n = 5 533; n = 4 594); frequency of not enough money (n = 5 543; n = 4 597); difficulty paying bills (n = 5 549; n = 4 602).

Additional adjustment for other lifestyle variables had little effect on the measures of association either for general or central obesity (see Additional file 1: Table S4 and Table S5).

## Discussion

### Synopsis of results

This cross-sectional, population-based study of UK over-50s showed social class, education, and housing tenure gradients in obesity. It further demonstrated strong associations with obesity for three types of financial hardship that were independent of social class, education and housing tenure. Independent associations were particularly strong for central obesity in women compared to men.

### Methodological considerations

The financial hardship variables were self-reported and like all such variables may be subject to recall or social desirability bias. Interpretation of the meaning of financial hardship can also vary widely across the population; equivalent levels of financial strain can be perceived and experienced as a normative status of daily living for some groups but as deprivation for others [19]. Precedent exists, however, for the measures used here as findings of independent associations are consistent with other studies of self-reported and objective health outcomes in similarly-aged groups [14,19,20]. Although financial hardship was assessed before anthropometry measurement, we could not ascertain the duration of, or transition in, hardship in relation to obesity as the survey was administered once. Thus, there may have been misclassification of exposures stemming from changes to participants’ hardship levels in the interval between assessment of financial circumstances and anthropometric measurement. Such misclassification would be non-differential since it was unlikely to have been related to our outcomes and hence would have biased results towards the null.

Our findings may also be subject to residual confounding in two ways. First, income was not collected in EPIC-Norfolk and thus we could not account for income-based differences. However, income is not consistently shown to have an impact on weight in older adults or for both sexes [36,37], and may not sufficiently reflect structural resources in our sample of older adults since they likely also use savings to fund their expenses [38]. Our study nevertheless examined six socioeconomic indicators separately and also included education, social class and housing tenure in analyses of financial hardship. Second, parity was not analysed but may have confounded or mediated our associations between socioeconomic factors and obesity [39,40].

Notwithstanding some limitations, the study’s strengths include a large sample size, sex-specific analyses, adjustment for multiple known confounders and lifestyle variables, and two objective obesity measures. Finally, this cohort had similar characteristics to the general UK population apart from fewer smokers and lack of ethnic diversity [23,28], and so findings could be generalised to other white European-origin older adults.

### Relationship to previous work

Our work is novel in at least three ways. First, we examined three separate financial hardship exposure measures and thus provide unique information on how different types of the financial hardship domain might be associated with higher prevalence of obesity [41]. We therefore add depth to previous studies which combined hardship questions into one summary indicator [8,10,14,15,42-45]. Second, our focus on over-50s contributes new knowledge in support of a healthy ageing agenda as no studies of economic strain and health in older populations have assessed obesity, to our knowledge [19,22]. Third and most notably, our study is clinically relevant. We used two objective measures of obesity recommended as separate predictors of health risk, particularly central adiposity [46], rather than self-reported weight which is prone to systematic bias from inaccuracy of height and underreporting and misclassification for obese categories [47,48].

Our finding that financial hardship showed independent associations with BMI is consistent with current evidence which notably comes from occupational cohorts [8,14,44]. The Helsinki Health Study of middle-aged employees, mostly women, reported increased odds of self-reported BMI for frequent financial hardship independent of conventional SES and early life factors [8], but our estimates were slightly larger. Unlike our work, that study of self-reported BMI included only age and no other BMI-related covariates associated with standard SES and obesity [49]. Another study of the same occupational cohort found a higher odds of weight gain (≥5 kg) with increasing frequency of economic difficulties after conventional SES adjustment, but again without accounting for smoking status, living arrangement or other health behaviours [10]. The Whitehall II study of financial hardship and coronary events in middle-aged men also found that a higher economic difficulties score was associated with a higher BMI and waist-hip ratio measured objectively, but the age-adjusted association did not account for conventional SES [14]. To our knowledge, no other studies of financial hardship have reported on central obesity for us to compare our findings.

Existing evidence supports the notion that social inequalities in obesity differ between the sexes with SES differences being associated more strongly and consistently with BMI in women [5-7,50]. Sex differences have also been reported among the few studies examining financial hardship and obesity, suggesting independent associations are stronger in men [8,10]. Notably, those results were reported in a younger population comprised of civil servants. The present study of a population-based sample of older British adults revealed contrasting results. Conventional SES proxies appeared to be more strongly and consistently associated with men’s higher odds of obesity which does not have a clear explanation. By contrast, the main associations between financial hardship and obesity were larger than SES and stronger in women. However, after adjusting for conventional SES, sex differences observed in the magnitude of associations depended on the type of hardship and obesity measure. This observed reversal between the sexes in the strength of socioeconomic disparities in obesity might point to gender-based differences in the experience of financial hardship. For example, men and women have differential vulnerabilities to financial hardship as women report not having enough money for food twice more often than men [51,52], and they also have differential power in intra-household economics and division of labour [53,54].

Several potential mediators might explain our finding of a link between financial hardship and obesity. Financial hardship is a powerful stressor and sociologists have shown the utility of coping and social support in explaining differences between socially and economically demarcated groups in effects of financial hardship [42,55]. Coping behaviours, involving the manipulation of goals and values, were found to be effective for minimizing adverse effects of household financial strain, and to be used to a greater extent by socially advantaged groups, namely men, the educated, and the affluent [55]. Other potential mediators include psychological resources such as self-esteem and sense of mastery [22]. Structural factors range from consumer prices of goods and services (e.g. food, transportation) [43], and neighbourhood access to healthy foods and safe spaces for physical activity [56], to employment [57,58] and cultural norms and social meanings reinforced through media and advertising [59,60]. Since financial hardship was a stronger correlate of obesity than conventional SES, excess weight and abdominal fat may be more directly influenced by mechanisms related to spending power and material resources, including lack of sleep from financial worries and physiological responses to hardship-related stress, than by non-material factors such as social roles, cultural norms, and knowledge. Both chronic stress and insufficient sleep have independent associations with obesity [61-63].

Although people of all ages may encounter financial hardship, adults in older age groups are at greater risk of increased financial hardship which commonly results from events they are more likely to experience such as divorce, death of spouse, or involuntary job loss [22,42]. Our results suggest that monetary and coping interventions may be useful in efforts to reduce obesity among over-50s. Formal mediation analyses of stress-related indicators are warranted to examine physiological mechanisms of influence between financial hardship and obesity in older women and men. Future research should also explore how both social and economic aspects of an individual’s life circumstances interact to produce combined effects on obesity as called for in the public health research and policy literature [64,65]. Nevertheless, prevention of obesity in over-50s would benefit more from an increased focus on their experience of financial hardship in addition to their education or income levels.

## Conclusions

British over-50s reporting greater levels of financial hardship were more likely to have excess weight and abdominal fat. Likelihood of obesity was more strongly correlated with financial hardship than conventional markers of SES. Thus, financial hardship indicators provided additional explanatory power beyond education, social class or home-ownership in understanding variation in prevalence of obesity in over-50 women and men. Our findings confirm that it is not sufficient to solely consider education, social class or home-ownership when examining the role of socioeconomic factors in the prevention of obesity or in weight support among older adults. Rather, public health policies and strategies need to support older people in terms of their more contemporaneous economic concerns. Interventions and practice standards to reduce or prevent obesity might include coping and monetary strategies and a focus on meeting bill payments might be a suitable target for approaches to address obesity.

## Competing interests

The authors declare that they have no competing interests.

## Authors’ contributions

AIC conceived the study, analysed the data, interpreted results and led writing of the manuscript. PM contributed to the study design, supervised the analysis, assisted in interpretation and contributed to the manuscript. NGF and MS informed the study design and analysis. PS designed and implemented the HLEQ Research Programme in the EPIC cohort; NJW is PI on the EPIC Norfolk study. All authors were involved in writing the paper and approved the final version.

## Pre-publication history

The pre-publication history for this paper can be accessed here:

http://www.biomedcentral.com/1471-2458/13/1039/prepub

## Supplementary Material

Additional file 1: Figure S1 The process of sample selection from the EPIC-Norfolk cohort. **Table S2**: Characteristics of self-reported financial hardship for over-50s in the EPIC-Norfolk cohort. **Table S3**: Odds ratios of general and central obesity in women and men (≥50 years) across levels of social class, education, and housing tenure in the EPIC-Norfolk cohort. **Table S4**: Odds ratio of general obesity across levels of financial hardship in women and men (≥50 years) in the EPIC-Norfolk cohort. **Table S5**: Odds ratio of central obesity across levels of financial hardship in women and men (≥50 years) in the EPIC-Norfolk cohort. Click here for file

## References

[B1] WHO Global health risks: mortality and burden of disease attributable to selected major risks 2009 Geneva: World Health Organization

[B2] Finkelstein EA Trogdon JG Brown DS Allaire BT Dellea PS Kamal-Bahl SJ The lifetime medical cost burden of overweight and obesity: implications for obesity prevention Obesity 2008 16 8 1843 1848 10.1038/oby.2008.290 18535543

[B3] Nolte E McKee M Caring for people with chronic conditions: a health system perspective 2008 Maidenhead, UK: Open University Press/McGraw Hill Education

[B4] Wakabayashi I Necessity of both waist circumference and waist-to-height ratio for better evaluaion of central obesity Metab Syndr Relat Disord 2013 11 3 189 94 10.1089/met.2012.0131 23451815

[B5] Pomerlau J Knai C Branca F Robertson A Rutter H McKee M Brunner E Consortium TE-P Tackling the social and economic determinants of nutrition and physical activity for the prevention of obesity across Europe: D3.1 Review of the literature of obesity (and inequalities in obesity) in Europe and of its main determinatns: nutrition and physical activity 2008 London, UK: London School of Hygiene& Tropical Medicine 1 55

[B6] McLaren L Socioeconomic status and obesity Epid Rev 2007 29 1 29 48 10.1093/epirev/mxm001 17478442

[B7] Langenberg C Hardy R Kuh D Brunner E Wadsworth M Central and total obesity in middle aged men and women in relation to lifetime socioeconomic status: evidence from a national birth cohort J Epidemiol Commun H 2003 57 10 816 822 10.1136/jech.57.10.816 PMC1732299 14573589

[B8] Laaksonen M Sarlio-Lähteenkorva S Lahelma E Multiple dimensions of socioeconomic position and obesity among employees: The Helsinki Health Study Obes Res 2004 12 11 1851 1858 10.1038/oby.2004.230 15601982

[B9] Sutton R Chapter 10: Adult anthropometric measures, overweight and obesit Healthy Survey for England - 2011, Health, social care and lifestyles. edn 2012 [online] http://www.hscic.gov.uk/catalogue/PUB09300: Health & Social Care Information Centre

[B10] Loman T Lallukka T Laaksonen M Rahkonen O Lahelma E Multiple socioeconomic determinants of weight gain: the Helsinki Health Study BMC Public Health 2013 13 259 266 10.1186/1471-2458-13-259 23517457 PMC3608219

[B11] Ouellette T Burstein N Long D Beecroft E Measures of material hardship: final report 2004 Washington: U.S. Department of Health and Human Services

[B12] Laaksonen E Lallukka T Lahelma E Ferrie JE Rahkonen O Head J Marmot MG Martikainen P Economic difficulties and physical functioning in Finnish and British employees: contribution of social and behavioural factors Eur J Public Health 2011 21 4 456 462 10.1093/eurpub/ckq089 20616102 PMC3139100

[B13] Laaksonen E Martikainen P Lallukka T Lahelma E Ferrie J Rahkonen O Marmot M Head J Economic difficulties and common mental disorders among Finnish and British white-collar employees: the contribution of social and behavioural factors J Epidemiol Commun H 2009 63 6 439 446 10.1136/jech.2008.077198 PMC2788762 19221110

[B14] Ferrie J Martikainen P Shipley M Marmot M Self-reported economic difficulties and coronary events in men: evidence from the Whitehall II study Int J Epidemiol 2005 34 3 640 648 15831564 10.1093/ije/dyi063

[B15] Lallukka T Ferrie JE Rahkonen O Shipley MJ Pietiläinen O Kivimäki M Marmot MG Lahelma E Change in economic difficulties and physical and mental functioning: evidence from British and Finnish employee cohorts Scand J Work Environ Health 2013 doi: 10.5271/sjweh.3366 10.5271/sjweh.3366 23609026

[B16] Rahkonen O Laaksonen M Karvonen S The contribution of lone parenthood and economic difficulties to smoking Soc Sci Med 2005 61 1 211 216 10.1016/j.socscimed.2004.11.044 15847973

[B17] Averett SL Smith JK Financial hardship and obesity: the link between weight and household debt Women 2013 100 100 100

[B18] Braveman P Cubbin C Egerter S Chideya S Marchi K Metzler M Posner S Socioeconomic status in health research: one size does not fit all JAMA 2005 294 22 2879 2888 10.1001/jama.294.22.2879 16352796

[B19] Kahn JR Pearlin LI Financial strain over the life course and health among older adults J Health Soc Behav 2006 47 1 17 31 10.1177/002214650604700102 16583773

[B20] Lallukka T Ferrie JE Kivimäki M Shipley MJ Rahkonen O Lahelma E Economic difficulties and subsequent sleep problems: evidence from British and Finnish occupational cohorts Sleep Med 2012 13 6 680 685 10.1016/j.sleep.2011.10.036 22445231 PMC3382711

[B21] Office of National Statistics Family spending 2011 edition: a report on the living costs and food survey 2010 2011 London: The Stationery Office

[B22] Pudrovska T Schieman S Pearlin LI Nguyen K The sense of mastery as a mediator and moderator in the association between economic hardship and health in late life J Aging Health 2005 17 5 634 660 10.1177/0898264305279874 16177454

[B23] Day N Oakes S Luben R Khaw KT Bingham S Welch A Wareham N EPIC-Norfolk: study design and characteristics of the cohort. European Prospective Investigation of Cancer Br J Cancer 1999 80 1 95 103 10466767

[B24] Surtees PG Wainwright NWJ The shackles of misfortune: social adversity assessment and representation in a chronic-disease epidemiological setting Soc Sci Med 2007 64 1 95 111 10.1016/j.socscimed.2006.08.013 16997441

[B25] Surtees PG Wainwright NWJ Brayne C Psychosocial aetiology of chronic disease: a pragmatic approach to the assessment of lifetime affective morbidity in an EPIC component study J Epidemiol Community Health 2000 54 2 114 122 10.1136/jech.54.2.114 10715744 PMC1731622

[B26] McFadden E Luben R Wareham N Bingham S Khaw K-T Occupational social class, risk factors and cardiovascular disease incidence in men and women: a prospective study in the European Prospective Investigation of Cancer and Nutrition in Norfolk (EPIC-Norfolk) cohort Eur J Epidemiol 2008 23 7 449 458 10.1007/s10654-008-9262-2 18509727

[B27] Pollack CE Chideya S Cubbin C Williams B Dekker M Braveman P Should health studies measure wealth?: a systematic review Am J Prev Med 2007 33 3 250 264 10.1016/j.amepre.2007.04.033 17826585

[B28] Surtees PG Wainwright NWJ Khaw KT Obesity, confidant support and functional health: cross-sectional evidence from the EPIC-Norfolk cohort Int J Obes Relat Metab Disord 2004 28 6 748 758 10.1038/sj.ijo.0802636 15052281

[B29] Diana FI Su H Winters PC Liang H Association of workplace chronic and acute stressors with employee weight status: data from worksites in turmoil J Occup Environ Med 2010 52 Suppl 1 S34 20061885 10.1097/JOM.0b013e3181c88525 PMC2911135

[B30] Kouvonen A Stafford M De Vogli R Shipley MJ Marmot MG Cox T Vahtera J Väänänen A Heponiemi T Singh-Manoux A Negative aspects of close relationships as a predictor of increased body mass index and waist circumference: the Whitehall II study Am J Public Health 2011 101 8 1474 1480 10.2105/AJPH.2010.300115 21680928 PMC3134502

[B31] Brunner EJ Chandola T Marmot MG Prospective effect of job strain on general and central obesity in the Whitehall II study Am J Epidemiol 2007 165 7 828 837 10.1093/aje/kwk058 17244635

[B32] Kahn HS Williamson DF Stevens JA Race and weight change in US women: the roles of socioeconomic and marital status Am J Public Health 1991 81 319 323 10.2105/AJPH.81.3.319 2036117 PMC1405001

[B33] Bingham SA Gill C Welch A Day K Cassidy A Khaw K-T Sneyd M Key TJA Roe L Day NE Comparison of dietary assessment methods in nutritional epidemiology: weighed records v. 24 h recalls, food-frequency questionnaires and estimated-diet records Brit J Nutr 1994 72 04 619 643 10.1079/BJN19940064 7986792

[B34] Wareham NJ Jakes RW Rennie KL Mitchell J Hennings S Day NE Validity and repeatability of the EPIC-Norfolk physical activity questionnaire Int J Epidemiol 2002 31 1 168 174 10.1093/ije/31.1.168 11914316

[B35] StataCorp Stata Statistical Software: Release 12 2011 College Station, TX: StataCorp LP

[B36] Cawley J Moran J Simon K The impact of income on the weight of elderly Americans Health Econ 2010 19 8 979 993 19691093 10.1002/hec.1541

[B37] Yoon YS Oh SW Park HS Socioeconomic status in relation to obesity and abdominal obesity in korean adults: a focus on Sex differences Obesity 2006 14 5 909 919 10.1038/oby.2006.105 16855201

[B38] Irz X Fratiglioni L Kuosmanen N Mazzocchi M Modugno L Nocella G Shakersain B Traill WB Xu W Zanello G Sociodemographic determinants of diet quality of the EU elderly: a comparative analysis in four countries Public Health Nutr 2013 1 13 [Epub ahead of print] 10.1017/S1368980013001146 PMC10282353 23659466

[B39] Gunderson EP Childbearing and obesity in women: weight before, during, and after pregnancy Obstet Gynecol Clin North Am 2009 36 2 317 10.1016/j.ogc.2009.04.001 19501316 PMC2930888

[B40] Weng HH Bastian LA Taylor DH Jr Moser BK Ostbye T Number of children associated with obesity in middle-aged women and men: results from the Health and Retirement Study J Womens Health 2004 13 1 85 91 10.1089/154099904322836492 15006281

[B41] Turrell G Hewitt B Patterson C Oldenburg B Measuring socio-economic position in dietary research: is choice of socio-economic indicator important? Public Health Nutr 2003 6 02 191 200 12675962 10.1079/PHN2002416

[B42] Pearlin LI The sociological study of stress J Health Soc Behav 1989 30 September 241 256 2674272

[B43] Burns C Bentley R Thornton L Kavanagh A Reduced food access due to a lack of money, inability to lift and lack of access to a car for food shopping: a multilevel study in Melbourne Victoria Public Health Nutr 2011 14 06 1017 1023 10.1017/S136898001000385X 21338555

[B44] Lallukka T Laaksonen M Rahkonen O Roos E Lahelma E Multiple socio-economic circumstances and healthy food habits Eur J Clin Nutr 2006 61 6 701 710 17180154 10.1038/sj.ejcn.1602583

[B45] Lallukka T Lahelma E Rahkonen O Changes in economic difficulties and subsequent sickness absence: a prospective register-linkage study BMJ Open 2013 3 1 e002212 10.1136/bmjopen-2012-002212 23303901 PMC3549204

[B46] Burkhauser RV Cawley J Beyond BMI: The value of more accurate measures of fatness and obesity in social science research J Health Econ 2008 27 2 519 529 10.1016/j.jhealeco.2007.05.005 18166236

[B47] Klein S Allison DB Heymsfield SB Kelley DE Leibel RL Nonas C Kahn R Waist circumference and cardiometabolic risk: a consensus statement from shaping America's health: association for weight management and obesity prevention; NAASO, The Obesity Society; The American Society for Nutrition; and The American Diabetes Association Am J Clin Nutr 2007 85 5 1197 1202 17490953 10.1093/ajcn/85.5.1197

[B48] Janssen I Katzmarzyk PT Ross R Waist circumference and not body mass index explains obesity-related health risk Am J Clin Nutr 2004 79 3 379 384 14985210 10.1093/ajcn/79.3.379

[B49] Manios Y Panagiotakos DB Pitsavos C Polychronopoulos E Stefanadis C Implication of socio-economic status on the prevalence of overweight and obesity in Greek adults: the ATTICA study Health Policy 2005 74 2 224 232 10.1016/j.healthpol.2005.01.014 16153482

[B50] Mackenbach JP Stirbu I Roskam A-JR Schaap MM Menvielle G Leinsalu M Kunst AE Socioeconomic inequalities in health in 22 European countries N Engl J Med 2008 358 23 2468 2481 10.1056/NEJMsa0707519 18525043

[B51] Denton M Prus S Walters V Gender differences in health: a Canadian study of the psychosocial, structural and behavioural determinants of health Soc Sci Med 2004 58 12 2585 2600 10.1016/j.socscimed.2003.09.008 15081207

[B52] Bihan H Castetbon K Mejean C Peneau S Pelabon L Jellouli F Le Clesiau H Hercberg S Sociodemographic factors and attitudes toward food affordability and health are associated with fruit and vegetable consumption in a low-income French population J Nutr 2010 140 4 823 830 10.3945/jn.109.118273 20181785

[B53] Katz E The intra-household economics of voice and exit Fem Econ 1997 3 3 25 46 10.1080/135457097338645

[B54] Brines J Economic dependency, gender, and the division of labor at home Am J Sociol 1994 100 3 652 688 10.1086/230577

[B55] Pearlin LI Schooler C The structure of coping J Health Soc Behav 1978 19 March 2 21 649936

[B56] Kamphuis CBM Explaining socioeconomic inequalities in health behaviours: the role of environmental factors 2008 Rotterdam: Erasmus

[B57] Rodriguez Martin A Martinez J Novalbos Ruiz J Escobar Jimenez L Overweight and obesity: the role of education, employment and income in Spanish adults Appetite 2008 51 2 266 272 10.1016/j.appet.2008.02.021 18406494

[B58] Rosmond R Björntorp P Psychosocial and socio-economic factors in women and their relationship to obesity and regional body fat distribution Int J Obes 1999 23 2 138 145 10.1038/sj.ijo.0800782 10078847

[B59] Murcott A The sociology of food and eating 1993 Aldershot, UK: Gower Publishing Company Ltd.

[B60] Lumbers M Raats M Shepherd R, Raats M Food choices in later life The Psychology of Food Choice. Volume Frontiers in Nutritional Science 2006 3 Wallingford, UK: CABI Publishing 280 310

[B61] Bose M Olivan B Laferrere B Stress and obesity: the role of the hypothalamic-pituitary-adrenal axis in metabolic disease Curr Opin Endocrinol Diabetes Obes 2009 16 5 340 346 10.1097/MED.0b013e32832fa137 19584720 PMC2858344

[B62] Cappuccio FP Taggart FM Kandala N-B Currie A Peile E Stranges S Miller MA Meta-analysis of short sleep duration and obesity in children and adults Sleep 2008 31 5 619 626 18517032 10.1093/sleep/31.5.619 PMC2398753

[B63] Björntorp P Do stress reactions cause abdominal obesity and comorbidities? Obes Rev 2001 2 2 73 86 10.1046/j.1467-789x.2001.00027.x 12119665

[B64] Killoran A Kelly M Evidence based public health: effectiveness and efficiency 2010 Oxford, UK: Oxford University Press

[B65] Kelly M Social differences in populations and their implications for public health Perspect Public Health 2009 129 5 207 208 10.1177/1757913909343889 19788159

