# Supplementary material for: Socioeconomic status, financial hardship and measured obesity in older adults: a cross-sectional study of the EPIC-Norfolk cohort
Source: BMC Public Health. 2013 Nov 4;13:1039. doi: 10.1186/1471-2458-13-1039 (PMC4228357; doi:10.1186/1471-2458-13-1039)
Supplement: Additional file 1: Figure S1 — The process of sample selection from the EPIC-Norfolk cohort. Table S2: Characteristics of self-reported financial hardship for over-50s in the EPIC-Norfolk cohort. Table S3: Odds ratios of general and central obesity in women and men (≥50 years) across levels of social class, education, and housing tenure in the EPIC-Norfolk cohort. Table S4: Odds ratio of general obesity across levels of financial hardship in women and men (≥50 years) in the EPIC-Norfolk cohort. Table S5: Odds ratio of central obesity across levels of financial hardship in women and men (≥50 years) in the EPIC-Norfolk cohort. [file 1471-2458-13-1039-S1.docx]

**Socioeconomic status, financial hardship and measured obesity in older adults**

a cross-sectional study of the EPIC-Norfolk cohort

**AUTHORS & AFFILIATIONS**

1. **Annalijn I Conklin**^1, 2^

2. **Nita G Forouhi^1^**

3. **Marc Suhrcke^2, 3^**

4. **Paul Surtees**^4^

5. **Nicholas J Wareham**^1, 2^

6. **Pablo Monsivais**^2, 4^

^1^ MRC Epidemiology Unit, University of Cambridge, Institute of Metabolic Science, Cambridge, Box 285, Addenbrooke’s Hospital, Hills Road, CB2 0QQ, UK

^2^ UK Clinical Research Collaboration Centre for Diet and Activity Research (CEDAR), Institute of Public Health, Box 296, Forvie Site, Robinson Way, Cambridge, CB2 0SR, UK

^3^ Faculty of Medicine and Health Sciences, University of East Anglia, Norwich, NR4 7TJ, UK

^4^ Department of Public Health and Primary Care, Institute of Public Health, University of Cambridge, CB1 8RN, UK

**Figure S1. The process of sample selection from the EPIC-Norfolk cohort**

**
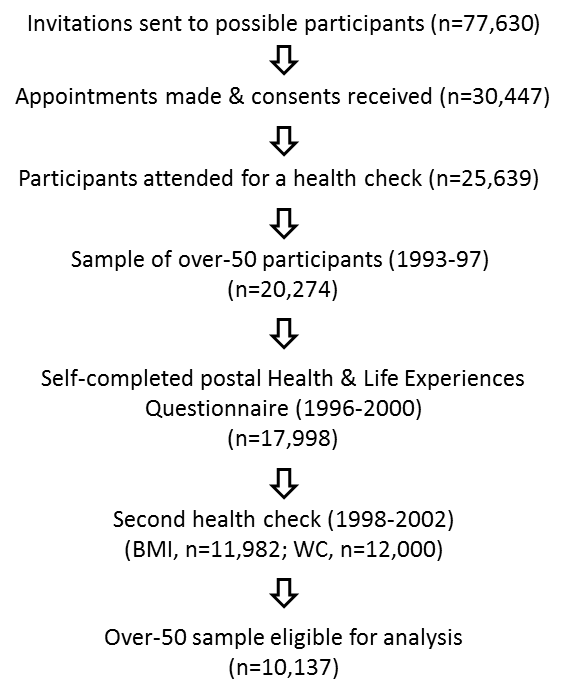
**

**Table S2. Characteristics of self-reported financial hardship for over-50s in the EPIC-Norfolk cohort**

|  | **Women** | **Degree-level education (highest)** | **Social class I & II**  **(highest)^1^** | **Home-owner** | **Regular car use** | **Poor/ moderate health** | **Ever smoker** | **Not married** | **General obesity^2^** | **Central obesity^2^** |
| --- | --- | --- | --- | --- | --- | --- | --- | --- | --- | --- |
| *Having enough money for needs (n=10 113)* | | | | | | | | | | |
| More than enough (n=1 934) | 53% | 26% | 67% | 99% | 18% | 10% | 47% | 16% | 13% | 21% |
| Just enough (n=7 220) | 55% | 11% | 43% | 92% | 22% | 17% | 52% | 20% | 18% | 28% |
| Less than enough (n=999) | 52% | 6% | 29% | 77% | 27% | 28% | 62% | 32% | 26% | 36% |
| *Frequency of not having enough money (n=10 126)* | | | | | | | | | | |
| Never (n=6 186) | 53% | 16% | 53% | 95% | 20% | 14% | 50% | 18% | 15% | 25% |
| Seldom (n=2 256) | 56% | 12% | 41% | 92% | 24% | 18% | 53% | 21% | 20% | 30% |
| Sometimes (n=1 223) | 59% | 6% | 32% | 84% | 26% | 22% | 53% | 24% | 21% | 32% |
| Often/ Always (n=461) | 57% | 8% | 30% | 72% | 28% | 31% | 59% | 35% | 25% | 35% |
| *Difficulty paying bills (n=10 137)* | | | | | | | | | | |
| None (n=6 118) | 55% | 15% | 51% | 95% | 21% | 14% | 51% | 18% | 16% | 25% |
| Very little (n=2 401) | 53% | 13% | 43% | 92% | 21% | 18% | 52% | 21% | 18% | 29% |
| Slight (n=741) | 56% | 10% | 39% | 86% | 24% | 22% | 55% | 22% | 22% | 32% |
| Some (n=731) | 59% | 8% | 32% | 77% | 26% | 29% | 54% | 33% | 25% | 34% |
| Great/ Very great (n=146) | 58% | 5% | 29% | 71% | 24% | 38% | 67% | 36% | 32% | 40% |

Note: Measurement time-points were: sex, education, class, accommodation (1993-1997); financial hardship measures and regular car use (1996-2000); general health, smoking status, marital status, and obesity (1998-2002). 1 Social class I = professional and class II = managerial and technical occupations based on Registrar General’s hierarchy. 2 General obesity (BMI≥30 kg/m2) was calculated from clinically measured height and weight; central obesity was calculated using sex-specific threshold criteria for clinically measured waist circumference (Women: ≥ 88cm; Men: ≥ 102cm).

**Table S3.** **Odds ratios of general and central obesity in women and men (≥50 years) across levels of social class, education, and housing tenure in the EPIC-Norfolk cohort**

|  | **General obesity** | |  | **Central obesity** | | |
| --- | --- | --- | --- | --- | --- | --- |
|  | **Women** | **Men** |  | **Women** | **Men** | |
| ***Social Class*** | *(n=4870)* | *(n=4006)* |  | *(n=4875)* | | *(n=4010)* |
| I | 1.00 | 1.00 |  | 1.00 | 1.00 | |
| II | 1.16  (0.83, 1.61) | 1.27  (0.86, 1.86) |  | 0.95  (0.72, 1.24) | 1.00  (0.76, 1.33) | |
| III–non manual | 1.09  (0.77, 1.55) | 1.63  (1.07, 2.49) |  | 0.88  (0.66, 1.17) | 1.15  (0.84, 1.59) | |
| III–manual | 1.41  (1.00, 2.00) | 1.61  (1.07, 2.40) |  | 1.04  (0.78, 1.39) | 1.09  (0.81, 1.47) | |
| IV | 1.76  (1.22, 2.53) | 1.42  (0.92, 2.19) |  | 1.19  (0.87, 1.61) | 1.17  (0.84, 1.61) | |
| V | 2.09  (1.32, 3.32) | 1.36  (1.29, 4.33) |  | 1.55  (1.03, 2.33) | 1.36  (0.81, 2.28) | |
| ***Education*** | *(n=4968)* | *(n=4057)* |  | *(n=4973)* | | *(n=4061)* |
| Degree | 1.00 | 1.00 |  | 1.00 | 1.00 | |
| A-level | 1.05  (0.81, 1.36) | 1.51  (1.12, 2.03) |  | 1.07  (0.86, 1.34) | 1.19  (0.95, 1.49) | |
| O-level | 1.13  (0.83, 1.56) | 1.14  (0.75, 1.73) |  | 0.96  (0.73, 1.27) | 1.22  (0.90, 1.67) | |
| No qualification | 1.42  (1.10, 1.84) | 1.88  (1.37, 2.56) |  | 1.31  (1.05, 1.64) | 1.51  (1.19, 1.91) | |
| ***Housing tenure*** | *(n=4522)* | *(n=3689)* |  | *(n=4527)* | | *(n=3693)* |
| Owner | 1.00 | 1.00 |  | 1.00 | 1.00 | |
| Renting, private | 1.35  (0.88, 2.09) | 1.43  (0.83, 2.45) |  | 1.33  (0.90, 1.96) | 1.18  (0.74, 1.89) | |
| Renting, public | 1.42  (1.05, 1.90) | 2.01  (1.37, 2.95) |  | 1.68  (1.29, 2.19) | 1.78  (1.26, 2.50) | |

Sex-specific odds ratios (95% confidence intervals) obtained by multivariable logistic regression analysis adjusting for age, marital status, smoking status, total energy intake, physical activity and energy expenditure, and total alcohol intake. General obesity (BMI ≥ 30kg/m^2^); Central obesity (women: waist circumference, WC≥88cm; men: WC≥102cm).

**Table S4. Odds ratio of general obesity across levels of financial hardship in women and men (≥50 years) in the EPIC-Norfolk cohort**

|  | **Women** | | | |  | **Men** | | | |
| --- | --- | --- | --- | --- | --- | --- | --- | --- | --- |
|  | ***Model A*** | ***Model B:***  ***A + education*** | ***Model C:***  ***B + social class*** | ***Model D:***  ***C + housing tenure*** |  | ***Model A*** | ***Model B:***  ***A + education*** | ***Model C:***  ***B + social class*** | ***Model D:***  ***C + housing tenure*** |
| *Enough money for needs* | | | | | | | | | |
| More than enough | 1.00 | 1.00 | 1.00 | 1.00 |  | 1.00 | 1.00 | 1.00 | 1.00 |
| Just enough | 1.44  (1.16, 1.78) | 1.36  (1.09, 1.69) | 1.33  (1.07, 1.66) | 1.33  (1.06, 1.66) |  | 1.04  (0.81, 1.33) | 0.98  (0.76, 1.26) | 0.94  (.73, 1.21) | 0.92  (0.71, 1.19) |
| Less than enough | 2.39  (1.77, 3.22) | 2.21  (1.63, 3.00) | 2.10  (1.54, 2.87) | 1.98  (1.44, 2.72) |  | 2.04  (1.46, 2.84) | 1.88  (1.35, 2.63) | 1.77  (1.26, 2.49) | 1.72  (1.21, 2.44) |
| *Frequency of not enough money* | | | | | | | | | |
| Never | 1.00 | 1.00 | 1.00 | 1.00 |  | 1.00 | 1.00 | 1.00 | 1.00 |
| Seldom | 1.35  (1.13, 1.62) | 1.32  (1.10, 1.59) | 1.31  (1.09, 1.58) | 1.30  (1.08, 1.56) |  | 1.29  (1.03, 1.62) | 1.27  (1.01, 1.59) | 1.25  (0.99, 1.57) | 1.26  (1.00, 1.59) |
| Sometimes | 1.55  (1.24, 1.93) | 1.50  (1.20, 1.86) | 1.48  (1.18, 1.85) | 1.41  (1.12, 1.77) |  | 1.36  (1.01, 1.82) | 1.31  (0.98, 1.76) | 1.30  (0.96, 1.74) | 1.23  (0.91, 1.67) |
| Often/ Always | 1.69  (1.21, 2.37) | 1.60  (1.14, 2.25) | 1.56  (1.11, 2.19) | 1.48  (1.04, 2.10) |  | 2.25  (1.50, 3.37) | 2.18  (1.45, 3.28) | 2.09  (1.37, 3.18) | 2.07  (1.35, 3.17) |
| *Difficulty paying bills* | | | | | | | | | |
| None | 1.00 | 1.00 | 1.00 | 1.00 |  | 1.00 | 1.00 | 1.00 | 1.00 |
| Very little | 1.35  (1.12, 1.62) | 1.33  (1.11, 1.60) | 1.30  (1.08, 1.57) | 1.31  (1.08, 1.57) |  | 1.03  (0.82, 1.29) | 1.02  (0.81, 1.28) | 1.02  (0.81, 1.29) | 1.00  (0.79, 1.26) |
| Slight | 1.75  (1.33, 2.30) | 1.74  (1.32, 2.29) | 1.74  (1.32, 2.29) | 1.67  (1.26, 2.21) |  | 1.05  (0.72, 1.52) | 1.04  (0.71, 1.51) | 1.04  (0.71, 1.51) | 1.03  (0.71, 1.51) |
| Some | 2.02  (1.55, 2.64) | 1.95  (1.49, 2.55) | 1.87  (1.43, 2.45) | 1.82  (1.38, 2.40) |  | 1.74  (1.23, 2.46) | 1.71  (1.21, 2.42) | 1.64  (1.16, 2.34) | 1.57  (1.09, 2.27) |
| Great/ Very great | 2.46  (1.44, 4.21) | 2.35  (1.37, 4.02) | 2.37  (1.38, 4.07) | 2.18  (1.24, 3.83) |  | 3.41  (1.79, 6.47) | 3.35  (1.76, 6.37) | 3.37  (1.76, 6.44) | 3.37  (1.74, 6.55) |

Sex-specific odds ratios (95% confidence intervals) for general obesity (BMI ≥ 30kg/m^2^) obtained by multivariable logistic regression analysis adjusting for age, marital status, smoking status, total energy intake, physical activity and energy expenditure, and total alcohol intake. Final numbers (Model D) of women and men, respectively, for money (n=4 380; n=3 606); frequency of insufficient money (n=4 387; n=3 605); difficulty paying bills (n=4 388; n=3 610).

**Table S5. Odds ratio of central obesity across levels of financial** **hardship in women and men (≥50 years) in the EPIC-Norfolk cohort**

|  | **Women** | | | |  | **Men** | | | |
| --- | --- | --- | --- | --- | --- | --- | --- | --- | --- |
|  | ***Model A*** | ***Model B:***  ***A + education*** | ***Model C:***  ***B + social class*** | ***Model D:***  ***C + housing tenure*** |  | ***Model A*** | ***Model B:***  ***A + education*** | ***Model C:***  ***B + social class*** | ***Model D:***  ***C + housing tenure*** |
| *Enough money for needs* | | | | | | | | | |
| More than enough | 1.00 | 1.00 | 1.00 | 1.00 |  | 1.00 | 1.00 | 1.00 | 1.00 |
| Just enough | 1.46  (2.21, 1.75) | 1.41  (1.17, 1.70) | 1.42  (1.18, 1.72) | 1.43  (1.18, 1.73) |  | 1.17  (0.96, 1.42) | 1.12  (0.91, 1.36) | 1.13  (0.92, 1.38) | 1.10  (0.90, 1.35) |
| Less than enough | 2.53  (1.94, 3.31) | 2.42  (1.85, 3.18) | 2.41  (1.83, 3.18) | 2.24  (1.69, 2.98) |  | 1.80  (1.35, 2.39) | 1.70  (1.27, 2.26) | 1.67  (1.24, 2.24) | 1.64  (1.21, 2.21) |
| *Frequency of not enough money* | | | | | | | | | |
| Never | 1.00 | 1.00 | 1.00 | 1.00 |  | 1.00 | 1.00 | 1.00 | 1.00 |
| Seldom | 1.35  (1.15, 1.59) | 1.33  (1.13, 1.57) | 1.32  (1.12, 1.56) | 1.32  (1.12, 1.55) |  | 1.31  (1.09, 1.57) | 1.29  (1.07, 1.55) | 1.29  (1.07, 1.56) | 1.29  (1.07, 1.56) |
| Sometimes | 1.48  (1.21, 1.80) | 1.45  (1.19, 1.77) | 1.44  (1.18, 1.76) | 1.38  (1.12, 1.69) |  | 1.49  (1.17, 1.88) | 1.45  (1.14, 1.84) | 1.46  (1.15, 1.86) | 1.43  (1.12, 1.83) |
| Often/ Always | 1.96  (1.45, 2.66) | 1.90  (1.40, 2.57) | 1.92  (1.41, 2.60) | 1.80  (1.31, 2.46) |  | 1.50  (1.03, 2.18) | 1.46  (1.00, 2.13) | 1.42  (0.96, 2.09) | 1.43  (0.96, 2.13) |
| *Difficulty paying bills* | | | | | | | | | |
| None | 1.00 | 1.00 | 1.00 | 1.00 |  | 1.00 | 1.00 | 1.00 | 1.00 |
| Very little | 1.38  (1.18, 1.62) | 1.37  (1.17, 1.61) | 1.34  (1.14, 1.58) | 1.35  (1.15, 1.60) |  | 1.15  (0.96, 1.38) | 1.14  (0.96, 1.37) | 1.16  (0.96, 1.39) | 1.14  (0.94, 1.37) |
| Slight | 1.48  (1.15, 1.91) | 1.47  (1.15, 1.90) | 1.49  (1.15, 1.92) | 1.42  (1.09, 1.84) |  | 1.36  (1.02, 1.82) | 1.35  (1.01, 1.81) | 1.35  (1.01, 1.81) | 1.37  (1.02, 1.84) |
| Some | 1.86  (1.45, 2.38) | 1.82  (1.42, 2.33) | 1.80  (1.40, 2.30) | 1.76  (1.37, 2.27) |  | 1.59  (1.18, 2.15) | 1.57  (1.16, 2.12) | 1.54  (1.13, 2.09) | 1.46  (1.06, 2.01) |
| Great/ Very great | 3.24  (1.97, 5.33) | 3.14  (1.91, 5.18) | 3.11  (1.87, 5.17) | 2.98  (1.77, 5.02) |  | 1.55  (0.81, 2.95) | 1.52  (0.80, 2.90) | 1.56  (0.81, 2.98) | 1.58  (0.81, 3.06) |

Sex-specific odds ratios (95% confidence intervals) obtained by multivariable logistic regression analysis adjusting for age, marital status, smoking status, total energy intake, physical activity and energy expenditure, and total alcohol intake. Central obesity for women (waist circumference, WC≥ 88cm) and for men (WC≥ 102cm). Final numbers (Model D) of women and men, respectively, for money (n=4 385; n=3 610); frequency of insufficient money (n=4 392; n=3 609); difficulty paying bills (n=4 393; n=3 614).
